# Proteomic analyses and identification of arginine methylated proteins differentially recognized by autosera from anti-Sm positive SLE patients

**DOI:** 10.1186/1423-0127-20-27

**Published:** 2013-05-04

**Authors:** Hong-How Chang, Huan-Hsuan Hu, Yu-Jen Lee, Hung-Ming Wei, Ming-Chun Fan-June, Tsai-Ching Hsu, Gregory J Tsay, Chuan Li

**Affiliations:** 1 Institute of Oral Biology, Chung Shan Medical University, Taichung, Taiwan, Republic of China; 2 Department of Biomedical Sciences, Chung Shan Medical University, No.110, Sec.1, Jian-guo N. Rd., Taichung, Taiwan 40201, Republic of China; 3 Institute of Biochemistry and Biotechnology, Chung Shan Medical University, Taichung, Taiwan, Republic of China; 4 Institute of Microbiology and Immunology, Chung Shan Medical University, Taichung, Taiwan, Republic of China; 5 Department of Medicine, Chung Shan Medical University Hospital, Taichung, Taiwan, Republic of China; 6 Department of Medical Research, Chung Shan Medical University Hospital, Taichung, Taiwan, Republic of China

**Keywords:** SLE, Anti-Sm, Arginine methylation, CNBP, hnRNP DL

## Abstract

**Background:**

Antibodies against spliceosome Sm proteins (anti-Sm autoantibodies) are specific to the autoimmune disease systemic lupus erythematosus (SLE). Anti-Sm autosera have been reported to specifically recognize Sm D1 and D3 with symmetric di-methylarginines (sDMA). We investigated if anti-Sm sera from local SLE patients can differentially recognize Sm proteins or any other proteins due to their methylation states.

**Results:**

We prepared HeLa cell proteins at normal or hypomethylation states (treated with an indirect methyltransferase inhibitor adenosine dialdehyde, AdOx). A few signals detected by the anti-Sm positive sera from typical SLE patients decreased consistently in the immunoblots of hypomethylated cell extracts. The differentially detected signals by one serum (Sm1) were pinpointed by two-dimensional electrophoresis and identified by mass spectrometry. Three identified proteins: splicing factor, proline- and glutamine-rich (SFPQ), heterogeneous nuclear ribonucleoprotein D-like (hnRNP DL) and cellular nucleic acid binding protein (CNBP) are known to contain methylarginines in their glycine and arginine rich (GAR) sequences. We showed that recombinant hnRNP DL and CNBP expressed in *Escherichia coli* can be detected by all anti-Sm positive sera we tested. As CNBP appeared to be differentially detected by the SLE sera in the pilot study, differential recognition of arginine methylated CNBP protein by the anti-Sm positive sera were further examined. Hypomethylated FLAG-CNBP protein immunopurified from AdOx-treated HeLa cells was less recognized by Sm1 compared to the CNBP protein expressed in untreated cells. Two of 20 other anti-Sm positive sera specifically differentiated the FLAG-CNBP protein expressed in HeLa cells due to the methylation. We also observed deferential recognition of methylated recombinant CNBP proteins expressed from *E. coli* by some of the autosera.

**Conclusion:**

Our study showed that hnRNP DL and CNBP are novel antigens for SLE patients and the recognition of CNBP might be differentiated dependent on the level of arginine methylation.

## Background

A common feature of autoimmune diseases such as systemic lupus erythematosus (SLE), rheumatoid arthritis (RA) and mixed connective tissue disease (MCTD) is the breakdown of tolerance to self antigens that leads to the production of antibodies reactive with multiple self proteins [1]. Many autoantigens are post-translationally modified. The modification might be induced by stresses such as inflammation, apoptosis, and aging. Inefficient clearance can lead to the presentation of the modified proteins by antigen presenting cells as novel foreign proteins. The possibility of posttranslational modification to create new self antigens or mask antigens normally recognized by the immune system has been proposed [2-4].

*N*^*G*^–monomethylarginine (MMA) and asymmetric *N*^*G*^*, N*^*G*^–dimethylarginines (aDMA) are frequently identified in various RNA binding proteins within the Arg Gly-Gly (RGG) context or Gly and Arg rich (GAR) region. The methylation is catalyzed by the type I protein arginine methyltransferase (PRMT) [5], [6]. Another type II PRMT modifies other methyl-accepting proteins such as myelin basic protein [7], core small nuclear ribonucleoprotein (SnRNP) Sm B/B′, D1, D3 [8], and the Sm-like proteins LSm4 [9] to form MMA and symmetric *N*^*G*^*, N′*^*G*^–dimethylarginine (sDMA).

Interestingly, several autoantigens of different autoimmune diseases are type I or type II methyl-accepting proteins. For example, fibrillarin (scleroderma) [10], hnRNP A1 (connective tissue diseases) [11], myelin basic protein (multiple sclerosis) [7], and SmD1 and D3 (SLE) [12] all contain methylarginines. Furthermore, peptides with aDMA modification were identified as natural MHC class I ligands, indicating that specific cytotoxic T-cell response against cells presenting aDMA modified peptides can be elicited [13].

Anti-Sm antibodies are directed against the common core proteins of U1, U2, U4 and U5 snRNP including SmB/B′, D1, D2, D3, E, F, G, with B/B′, D1 and D3 most frequently targeted. Anti-Sm antibodies are highly specific to SLE and are found in up to 30% of the SLE patients [14]. The direct link of arginine methylation and autoantibody recognition was shown by Brahms et al [8]. Ten of eleven different anti-Sm autosera recognized the sDMA-containing peptides of SmD1 and D3 but not unmethylated or aDMA-containing peptides [8]. We thus are interested in whether the anti-Sm sera from local SLE patients also preferentially recognize the methyl-modified Sm B/B′, D1 and D3 proteins, and if there might be other proteins that can be differentially recognized by the anti-Sm sera due to their methylation states. We treated HeLa cells with adenosine dialdehyde (AdOx), an indirect inhibitor of protein methylation, to accumulate hypomethylated proteins in cells. Among a few putative differentially recognized proteins, three proteins containing typical arginine and glycine (RG) sequences were identified. We showed that recombinant heterogeneous nuclear ribonucleoprotein D-like (hnRNP DL) and cellular nucleic acid binding protein (CNBP) expressed in *Escherichia coli* can be detected by anti-Sm positive sera. We provided evidences that arginine methylation of CNBP in the RG motif is critical for the recognition of the protein by some of the anti-Sm autosera.

## Methods

### Anti-Sm autosera from SLE patients

Anti-Sm autosera were collected from SLE patients from Division of Rheumatology, Department of internal medicine, Chung Shan Medical University Hospital, Taichung, Taiwan. All patients were followed up at Chung Shan Medical University Hospital and diagnosed using the 1982 revised criteria for SLE [15]. The presence of anti-Sm antibodies was tested by double diffusion method of ENA-1 kit (MBL, Nagoya, Japan) and direct antigen-specific ELISA kit (INOVA Diagnostics Inc., San Diego, CA, USA) as the manufacturer’s instructions. Most of the sera showed speckled or nucleolar type for the antinuclear antibody (ANA) test. The study was approved by the local Institutional Research Board. Human normal control and anti-Sm antibodies were purchased from INOVA Diagnostics (INOVA Diagnostics) and used as the control.

### Cell cultures and protein extraction

HeLa cell culture, methylation inhibitor treatment (adenosine dialdehyde, AdOx; Sigma-aldrich) and cell extract preparation and SDS-polyacrylamide gel electrophoresis (PAGE) were performed following the methods described in [16]. To prepare HeLa cell extracts for two-dimensional electrophoresis (2-DE), harvested cells were washed with phosphate buffer saline (PBS) then resuspended in rehydration buffer (8 M urea, 4% (w/v) CHAPS, 0.5% IPG buffer pH3–10 or 4–7, 60 mM dithiothreitol, 0.002% bromophenol blue). After 3-min shaking, cells were incubated on ice for five minutes then centrifuged at 12,000×g for 20 min at 4°C. Proteins in the extracts were quantified by BCA kit (Pierce) or 2-D quant kit (GE-Amersham Biosciences) with bovine serum albumin as the standard.

### Two-dimensional gel electrophoresis

HeLa cell extract protein (250 μg) was applied to immobilized pH gradient (IPG) strips (pH3–10 or 4–7, 7 cm) for isoelectrofocusing (IEF) electrophoresis. IEF was carried out in an IPGphore system as instructed by the manufacturer (GE Amersham Biosciences). Upon completion of IEF (13,350 Volt-hours), the strips were equilibrated and subjected to the second dimensional SDS-PAGE as described [17]. The gels were stained with coomassie brilliant blue or SyproRuby (Molecular Probes).

### Western blotting

Protein samples separated by SDS-PAGE or 2-D electrophoresis were transferred to nitrocellulose membranes. The membranes were blocked in 5% skimmed dry milk in TTBS (10 mM Tris-HCl, pH = 7.5; 100 mM NaCl; 0.1% tween 20) for 30 min, incubated with primary antibodies (1:200 dilution for 7E6 antibody and 1:500 dilution for anti-CNBP from Abcam; 1:550 dilution for SYM10, 1:900 dilution for SYM11 and 1:900 dilution for ASYM24 from Upstate) at 4°C overnight, washed three times in TTBS, then incubated with secondary antibody (anti-mouse or rabbit IgG horse radish peroxidase conjugate from Sigma) for 1 h. Chemiluminescent detection was performed using the Supersignal kit (Pierce) or Western Blotting Luminol Reagent (Santa Cruz Biotechnology, Santa Cruz, CA) according to the manufacturer’s instructions. If the first antibody is human serum, the dilution will be 1:200 and the concentration of skimmed dry milk in the blocking solution and antibody solution will be increased to 7% and 0.7% respectively.

### Mass spectrometry

Desired protein spots were manually picked and in gel digestion was performed using the Montage In Gel Digset_zp_ Kit (Millipore) as described [17]. The trypsin-digested peptides were then extracted, captured and eluted. The eluted peptides were vacuum-dried and analyzed by Core Facilities for Proteomics Research in the Institute of Biological Chemistry, Academia Sinica. Protein spots were subjected to concerted MALDI peptide mass fingerprinting (PMF) and CID MS/MS analysis for protein identification using a dedicated Q-Tof Ultima™ MALDI instrument (Micromass, Manchester, UK). The instrument systems were operated under MassLynx 4.0 and raw MS data were processed for database searching using ProteinLynx Global Server 2.0. For MALDI MS and MS/MS analysis, samples were premixed 1:1 with matrix solution (5 mg/ml CHCA in 50% acetonitrile, 0.1% v/v TFA and 2% w/v ammonium citrate). Within each well, as many parent ions meeting the predefined criteria (any peak within the *m/z* 800–3000 range with intensity above 10 count ± include/exclude list) will be selected for CID MS/MS using argon as collision gas and a mass dependent ±5 V rolling collision energy until end of probe pattern was reached, starting from the most intense peak. The LM and HM resolution of the quadrupole were both set at 10 to give a precursor selection window of about 4 Da wide.

### Protein identification

The MS or MS/MS spectra data were analyzed by Mascot mass fingerprinting and MS/MS ion search (http://www.matrixscience.com) with the following characteristics: Peptide mass fingerprinting: Database: NCBInr; Taxonomy: Homo sapiens; Enzyme: Trypsin; Fixed modification: Carbamidomethyl (C); Variable modifications: Oxidation (M); Max missed cut: 1; Peptide tolerance: 50 ppm; Mass values: MH^+^. MS/MS ion search: Database: NCBInr; Taxonomy: Homo sapiens; Enzyme: Trypsin; Max missed cut: 1; fixed modification: Carbamidomethyl (C); Variable modifications: Oxidation (M); Peptide tolerance: 50 ppm; fragment mass tolerance: 0.25 Da; Peptide charge: 1+; Data format: Micromass (.PKL); Instrument: MALDI-QUAD-TOF.

### Cloning of CNBP and plasmid constructs

cDNA clones of hnRNPDL and β-CNBP were obtained from Source BIOScience LifeSciences (Berlin, Germany). The coding region of hnRNP DL was amplified by polymerase chain reaction (PCR) with the hnRNP DL forward (5^′^-AAACCCGGGTATGGAGGATATGAACGAG-3^′^) and reverse (5^′^-AAAAGCGGCCGCTTTAGTATGGCTGGTAA-3′) primers and subcloned into a TA vector. The *SmaI-NotI* restriction fragment was subcloned into the pGEX4T vector to prepare GST-fused hnRNP DL. The coding region of CNBP was amplified with the CNBP forward primer (5^′^-AAGGATCCATGAGCAGCAATGAGT-3^′^) and reverse primer (5^′^-AAAAGCGGCCGCAATTAGGCTGTAGCCTCA-3^′^), and subcloned into a TA vector. The *BamH1-EcoRI* restriction fragments were subcloned into the pGEX4T vector to prepare GST-tagged CNBP. The CNBP coding sequence amplified by the primer set ZNF9-Not-F (5^′^-AAGCGGCCGCCATGAGCAGCAATG-3^′^) and ZNF9-*Bam*H-R (5^′^- AGGATCCAATTAGGCTGTAGCCTCA-3^′^) was cleaved and the 5^′^*Not*I and 3^′^*Bam*HI restriction fragment was subcloned into the pFLAG-CMV2 vector. The RG region deletion was created by QuikChange® II Site-Directed Mutagenesis Kit (Stratagene) with the primers (Znf9-DL1F: 5^′^- CTACTGGTGGAGGCTTCCAGTTTGTTTCC-3^′^ and Znf9-DL1R:5^′^- GAAACAAACTGGAAGCCTCCACCAGTAGG-3^′^) that contain the 33 bp deletion. The CNBP coding sequence was subcloned into pET28b by PCR amplification and *Bam*HI-*Sal*I restriction digestion.

### Purification of recombinant proteins expressed in *E. coli*

Expression of GST-CNBP or GST-hnRNP DL fusion proteins in *Escherichia coli* DH5αor BL21 (DE3) cells was induced with IPTG and purified using Glutathione Sepharose affinity chromatography (GE Amersham Biosciences) according to the manufacturer’s instructions. (His)_6_-tagged CNBP protein was prepared from *E. coli* cells transformed with pET-28b-CNBP. The pellet of the extract containing (His)_6_-fusion proteins was resuspended with 3 ml Buffer A (6 M Guanidine-HCL, 0.1 M NaH_2_PO_4_, 0.01 M Tris-HCL, 0.1 M β-mercaptoethanol, 0.01 M PMSF, pH 8.0) at room temperature for 1 hr, followed by centrifugation at 10000 × g for 10 min. The supernatant was loaded to a Ni-NTA agarose column (Qiagen). Bound (His)_6_-CNBP fusion proteins were washed and eluted according to the suggestions of the manufacturer. To prepare methylated CNBP, pET-28b-CNBP and pGEX-PRMT1 were co-transformed into BL21 (DE3) cells and selected with both ampicillin and kanamycin.

### Transfection and purification of FLAG-CNBP

For transfection, HeLa cells were transfected with pFLAG-CNBP plasmid using Lipofectamine (Invitrogen, Camarillo, CA). AdOx (20 μM) was included in the MEM medium 24 hr after transfection, and the cells were cultured for another 24 hr. HeLa cell extracts preparation and immunopurification of FLAG-CNBP protein were conducted as described [18].

## Results

### Proteins other than Sm proteins were differentially recognized by local SLE anti-Sm positive autosera due to their methylation status

**Figure 1:**
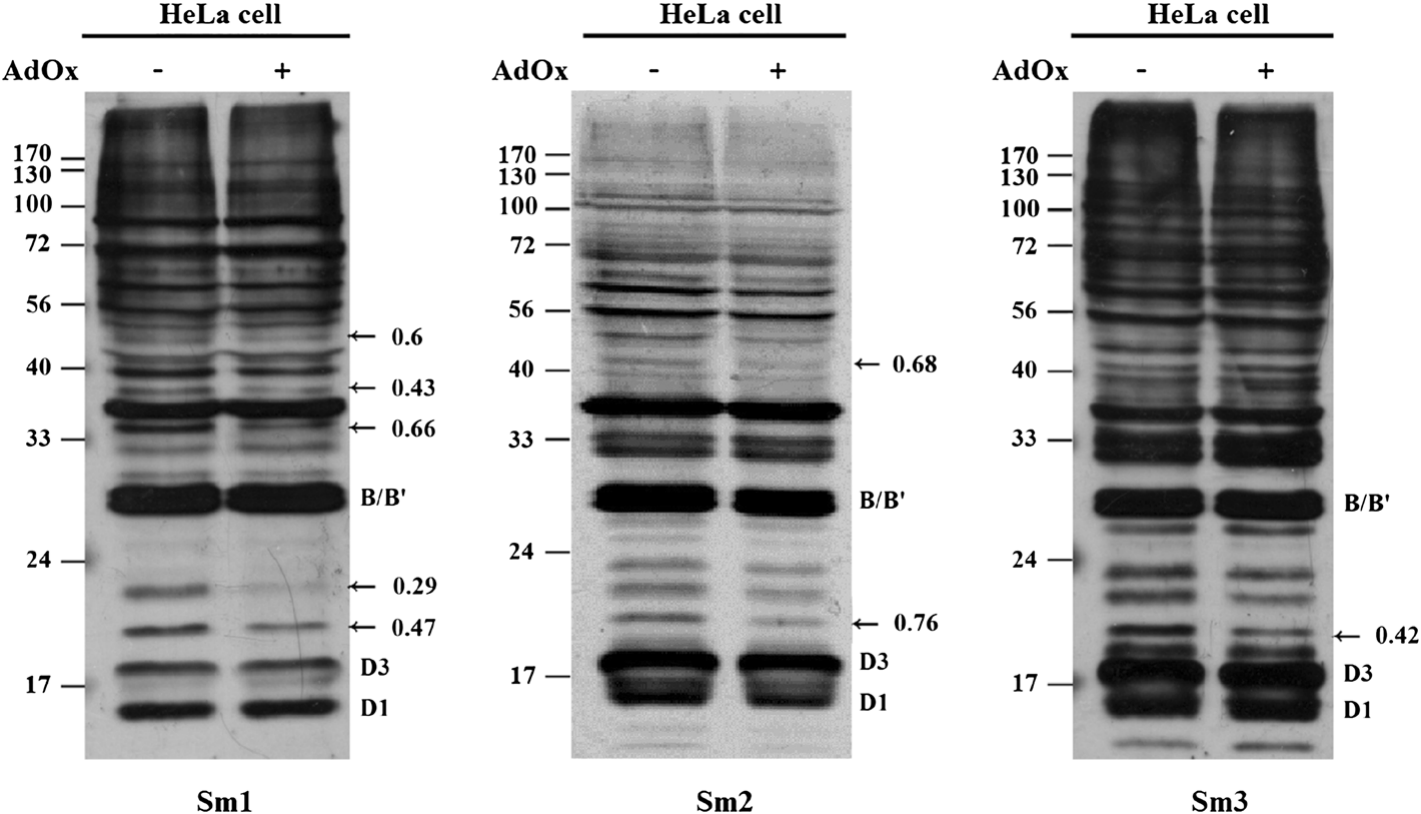
**Differential recognition of proteins due to methylation status by anti-Sm autosera.** HeLa cell extracts (30 μg of total protein) were prepared from cells grown in the presence or absence of 20 μM of AdOx. The cell extracts were separated by SDS-PAGE and transferred onto nitrocellulose membrane. Western blot analysis was performed using Sm1, Sm2 and Sm3 autosera. The positions of the differentially detected signals are indicated by arrows and the intensity ratios are shown. The positions of typical anti-Sm recognized SmB/B′, SmD1 and D3 proteins are also indicated.

We first examined whether the anti-Sm positive autosera from local SLE patients can differentially recognize Sm proteins or certain other proteins due to their methylation status. We prepared hypomethylated cell extracts from HeLa cells treated with the methyltransferase inhibitor AdOx. Reduced protein arginine methylation under the condition has been determined previously [8] and was examined for different batches of extracts prepared in this study (data not shown). For the pilot study, we included anti-Sm positive sera (Sm1, 2 and 3) from three typical SLE patients for immunoblot analyses. The overall detection patterns by the same serum were similar whether the proteins were from AdOx-treated or untreated HeLa cells. Signals correspond to SmB/B′ and Sm D1/D3 were detected clearly but without differential recognition. Nevertheless, reduced signals between molecular mass of 18 to 23 kDa were consistently detected by Sm1, 2 and 3 in the hypomethylation samples (Figure 1). The Sm1 serum appeared to differentially recognize more polypeptides at the molecular mass of about 36, 40 and 49 kDa.

**Figure 2:**
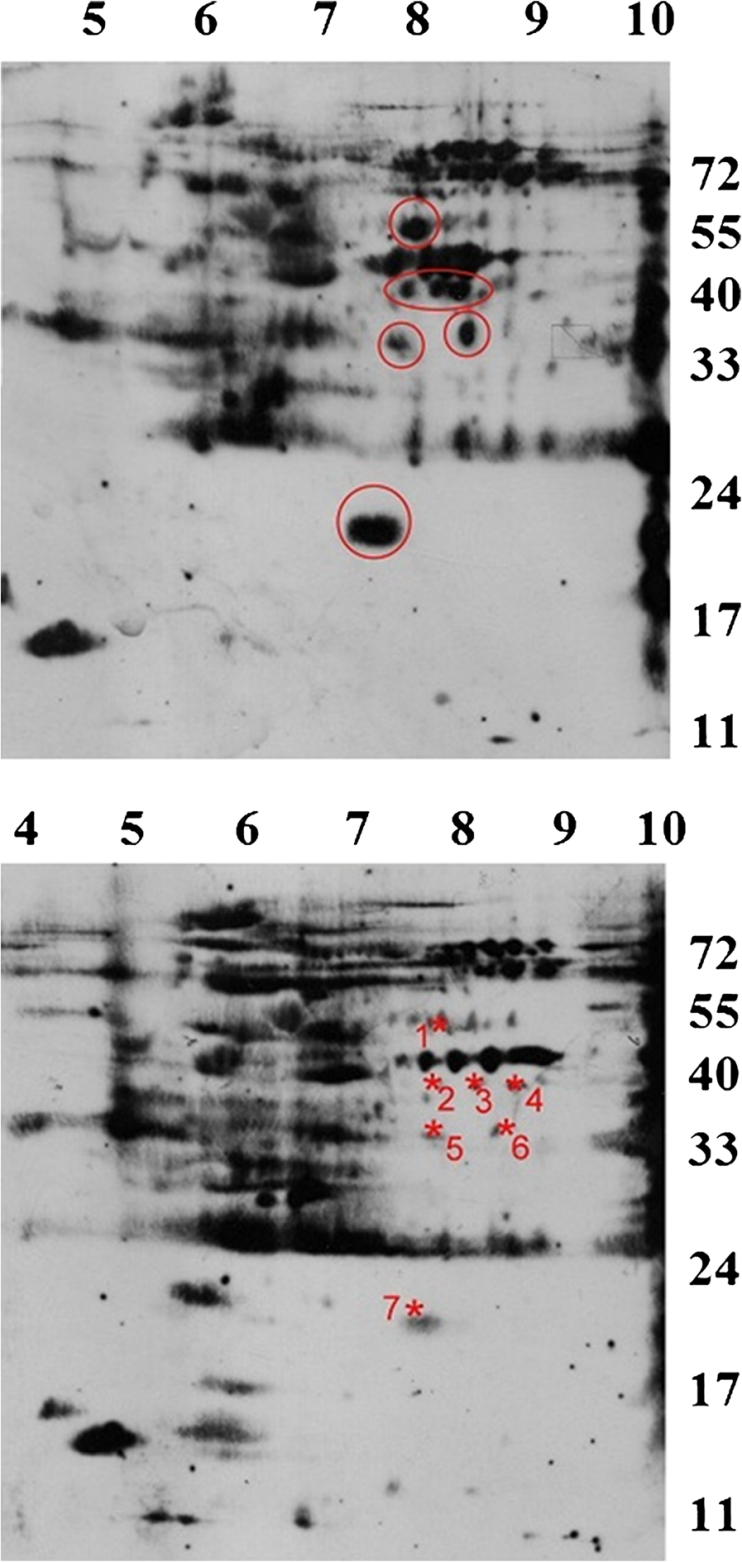
**Resolving differentially recognized proteins by Sm1 autosera due to methylation status by 2-DE.** HeLa cell extracts (250 μg of total protein) treated with AdOx (lower panel) or not (upper panel) were resolved by 2-DE, blotted and detected by anti-Sm1. The other parallel gel was stained by SyproRuby. The differentially detected signals that can match to protein spots are indicated by red asteroid and numbered.

To obtain better resolution of the putative methylarginine-dependent recognition targets of Sm1, we conducted two-dimensional electrophoresis (2-DE) to separate the HeLa cell proteins. Specific spots were strongly recognized by Sm1 from cell extracts with no AdOx treatment but not the extracts treated with AdOx (Figure 2). The spots were of similar molecular masses as those detected by one-dimensional SDS-PAGE. The differentially recognized spots by Sm1 in the immunoblots were compared with the protein spots of a parallel coomassie-stained 2-DE gel. The matched protein spots were digested with trypsin and subjected to mass spectrometry. Through proteomic analyses, seven putative differentially recognized spots by Sm1 were identified as eight different proteins. The identified polypeptides are listed in Table 1. For spot 1, 2 and 3, two different polypeptides were identified for the same spot. α-enolase (or called non-neuronal enolase) was identified for spot 2, 3, and 4 in a row with similar molecular masses but different isoelectric points (pIs). The unambiguous identification of a polypeptide should be supported by at least one peptide with a Mascot ion score higher than the number that indicates identity.

**Table 1:** Putative differentially recognized polypeptides by the Sm1 serum determined by mass spectrometry

**Spot number**	**Protein name/gene name**	**Accession number**	**Measured MW/pI**	**Theoretical MW/pI**	Mascot score (PMF/MS/MS)*	**Arginine methylation sites (predicted or experimental)**
1	Succinyl-CoA:3-ketoacid coenzyme A transferase 1, mitochondrial, OXCT1	P55809	53/7.8	56578/7.14	64/173	None
	Aldehyde dehydrogenase X, mitochondrial, ALDH1B1	P30837; B2R8F0; Q8WX76; Q9BV45		57626/6.36	88/34	None
2	Alpha-enolase, ENO1	GMP2; Q71V37; Q7Z3V6; Q8WU71; Q9UCH6; Q9UM55	40/7.7	47481/7.01	x /97	None
	Splicing factor, proline- and glutamine-rich, SFPQ	P23246; P30808; Q5SZ71		76216/4.95	x/52	RFRSRGGGGGFHRRG GGGGRGG (7, 9, 19, 25;) [19]; EEERRRRE (571); RGMGPGTPAGYGRG (681,693) [20]
3	Putative beta-actin-like protein 3, POTEKP	Q9BYX7; Q562N5	40/8.1	41989/5.91	x/95	None
	Alpha-enolase, ENO1			47481/7.01	x/84	None
4	Alpha-enolase, ENO1		39/8.3	47481/7.01	99/202	None
5	LIM and SH3 domain protein 1, LASP1	Q14847; Q96ED2; Q96IG0	33/7.8	30097/6.61	(50)/149	None
6	Heterogeneous nuclear ribonucleoprotein D-like, HNRNPDL	O14979; Q6SPF2; Q7KZ74; Q7KZ75; Q96IM0; Q96S43	34/8.3	46580/9.59	none/55	STYGKASRGG GNHQ (408) [19]
7	Cellular nucleic acid-binding protein, CNBP	Q5U0E9; Q6PJI7; Q96NV3	19/7.9	20704/8.00	x/56	TGGGRGRGMR SRGRGGFTSD RGFQFVSSSL [20]

*x indicates that the PMF search results were not consistent with the MS/MS results. “None” indicates that none of the PMF (peptide mass fingerprint) results (protein scores) are significant. The protein score in parentheses is not significant.

### Identification of known methylarginine containing proteins as the differentially recognized proteins

Three of the identified target proteins: splicing factor, proline- and glutamine-rich (SFPQ), heterogeneous nuclear ribonucleoprotein D-like (hnRNP DL or JKTBP1) and cellular nucleic acid-binding protein (CNBP), contain typical arginine and glycine rich sequences for arginine methylation. Arginine methylation of RNA binding proteins SFPQ and hnRNP D-like was reported by Ong *et al.*[19] and Uhlmann *et al.*[20] in proteomic screening of methylarginine containing proteins. A peptide (FGQGGAGPVGGQGPR) with a significant ion score of 54 (higher than 43 indicate identity) unambiguously identified the protein SFPQ in spot 2. However, this spot appeared to contain protein α-enolase also. The theoretical molecular weight and pI value of α-enolase is close to the experimental ones while the theoretical values of SFPQ are very different from the experimental ones. It is thus likely that the spot contained degradation products or small isoforms of SFPQ.

An RNA binding protein hnRNP DL with high sequence similarity with hnRNP D (AUF1) and hnRNP A1 shuttles between nucleus and cytoplasm [21]. Anti-hnRNP A1 has been reported in autoimmune diseases including RA, SLE and MCTD as reviewed [22]. Anti-hnRNP D antibodies were detected in SLE and RA patients [23]. However, autoantibody to hnRNP DL has not been reported.

**Figure 3:**
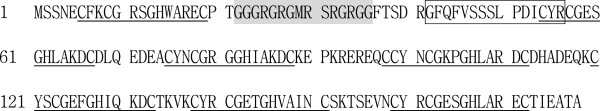
**The amino acid sequence of human CNBP.** The seven Cys-Cys-His-Cys (CCHC) type zinc knuckle domains (C-Φ-X-C-G-X3-H-X4-C, where Φis an aromatic amino acid and X is a variable amino acid) in the human CNBP protein are underlined. The RG sequence between zinc finger ZF1 and ZF2 is indicated with shading. The peptide sequence identified by MS/MS is boxed.

Another polypeptide of molecular mass about 19 kDa and pI 7.9 was identified by mascot search of the MS/MS data. A peptide (GFQFVSSSLPDICYR + carbamidomethyl) with significant mascot score 62 (scores greater than 39 indicate identity) identified the cellular nuclear acid binding protein (CNBP or ZNF9). CNBP contains seven tandem Cys-Cys-His-Cys (CCHC) type zinc knuckle domains and typical arginine and glycine (RG) sequences between the first and the second zinc buckle (Figure 3). CNBP was identified as a putative symmetric dimethylarginine containing protein in an immunopurification study with a symmetric dimethylarginine specific antibody SYM10 for methylarginine protein complexes [24]. Methylation at the arginine residues in the RG region of a specific splicing isoform was recently reported by Uhlmann *et al.*[20].

### Recognition of hnRNP DL and CNBP by other anti-Sm positive patient sera

**Figure 4:**
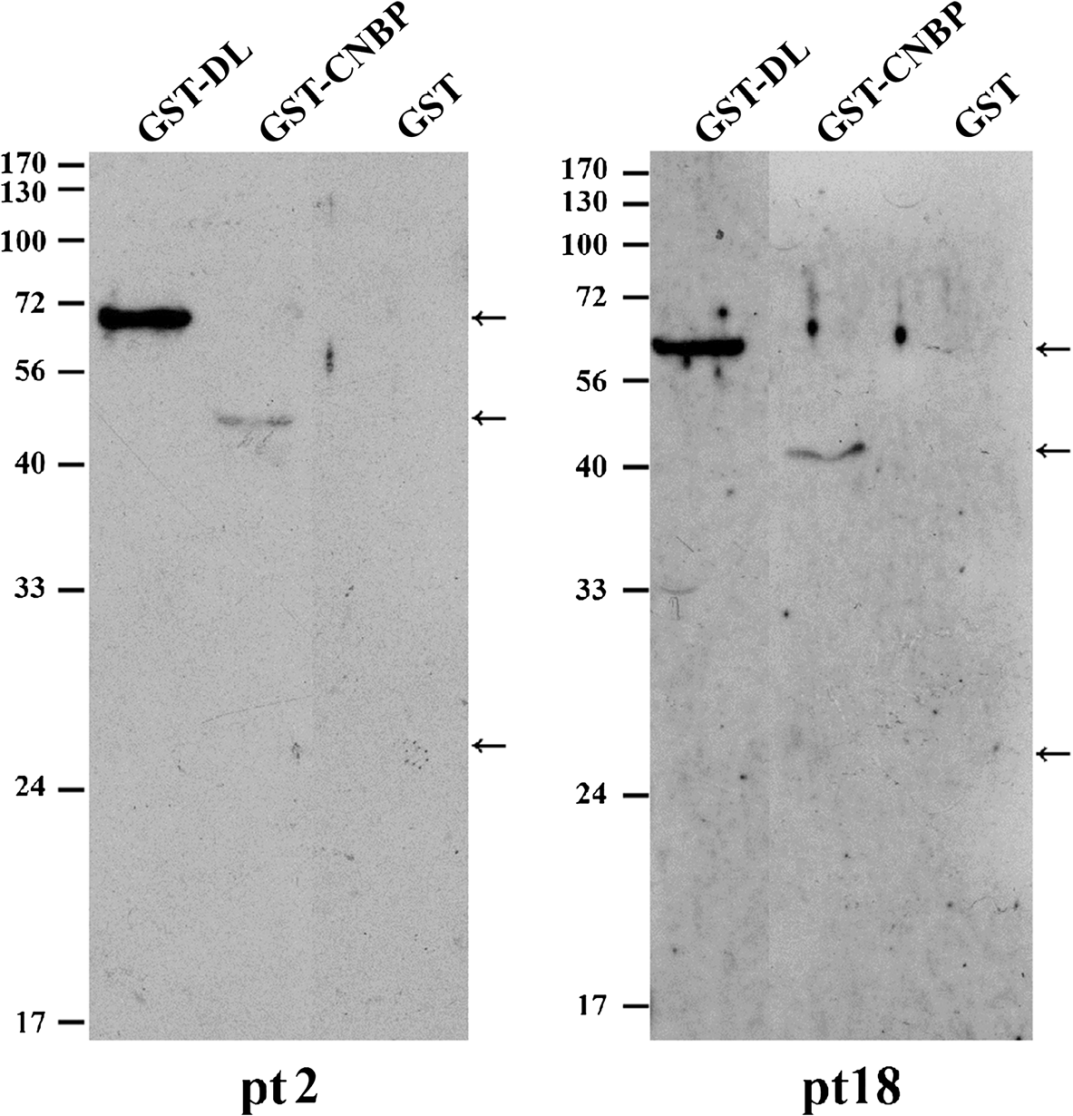
**Recognition of hnRNP DL and CNBP by anti-Sm positive SLE patient sera.** GST-fused hnRNP DL and CNBP were prepared as described in Methods. The GST-fused proteins or GST were applied to SDS-PAGE and the immunoblots detected by the autosera from patient (pt) 2 and 18 are shown.

We are interested in whether the RGG or GAR-containing proteins identified in this study are the targets for more SLE patients. We prepared recombinant GST-fused hnRNP DL and CNBP protein expressed in *Escherichia coli*. Besides the sera used in the pilot study, we included twenty anti-Sm positive sera from local SLE patients. Detections of the two putative autoantigens were summarized in Table 2. All of the sera can detect GST-tagged hnRNP DL and 60% (12/20) of the sera can detect CNBP by immunoblots. None of the sera can detect GST protein alone at the same protein amount. Part of the immunoblotting results were shown in Figure 4. Normal control sera pooled from individuals without autoimmunity marginally recognized the protein upon longer exposure (data not shown).

**Table 2:** Summery of the detection of recombinant hnRNP DL /CNBP and differential recognition of methylated CNBP protein by the anti-Sm positive sera from SLE patients screened in this study

**SLE patient**	**Detection of recombinant**			**Differential CNBP methylation**	
	**GST-hnRNP DL**	**GST-CNBP**	**(His)**_**6**_**-CNBP**	**FLAG-CNBP**	**(His)**_**6**_**-CNBP**
X1	+	+	+	−	+
X2	+	+	+	−	−
X3	+	−	+	−	±
X4	+	−	+	−	−
X5	+	+	+	+	±
X6	+	+	+	−	−
X7	+	−	+	−	−
X8	+	+	+	−	−
X9	+	+	+	−	−
X10	+	−	+	−	−
X11	+	+	+	−	±
X12	+	+	+	−	+
X13	+	−	±	−	−
X14	+	−	±	−	−
X15	+	+	+	−	−
X16	+	−	+	−	−
X17	+	+	+	−	±
X18	+	+	+	+	±
X19	+	−	+	−	+
X20	+	+	ND	−	ND

We further prepared recombinant (His)_6_-CNBP fusion proteins from *E. coli* cells and then screened Sm-positive sera for their recognition of recombinant CNBP. Basically, all of the sera examined, even the ones that did not detect GST-tagged CNBP, recognized (His)_6_-tagged CNBP. The results were listed in Table 2.

Anti-nuclear antibody (ANA) is critical for the diagnosis of SLE patients. We determined the ANA pattern of CNBP using a commercially available goat anti-CNBP antibody. Predominant nuclear positive staining was detected on standard HEp-2 cells at 100× dilution for the ANA test (data not shown) and similar nuclear expression pattern of CNBP was also detected in HeLa cells (Wei *et al.*, manuscript in preparation).

### Confirmation of the differential recognition of CNBP by Sm1 autosera due to arginine methylation

As the signal corresponding to CNBP was likely to be differentially recognized by Sm1, 2, and 3, we determined to confirm whether protein arginine methylation of CNBP is critical for the differentially recognition. The protein stain of the CNBP spots of AdOx-treated or untreated cell extracts in the 2-DE gels were of similar level, suggesting that the differential recognition was not due to reduced level of CNBP protein (data not shown), and thus was more likely to be due to the reduced methylation level of CNBP in the AdOx-treated samples.

**Figure 5:**
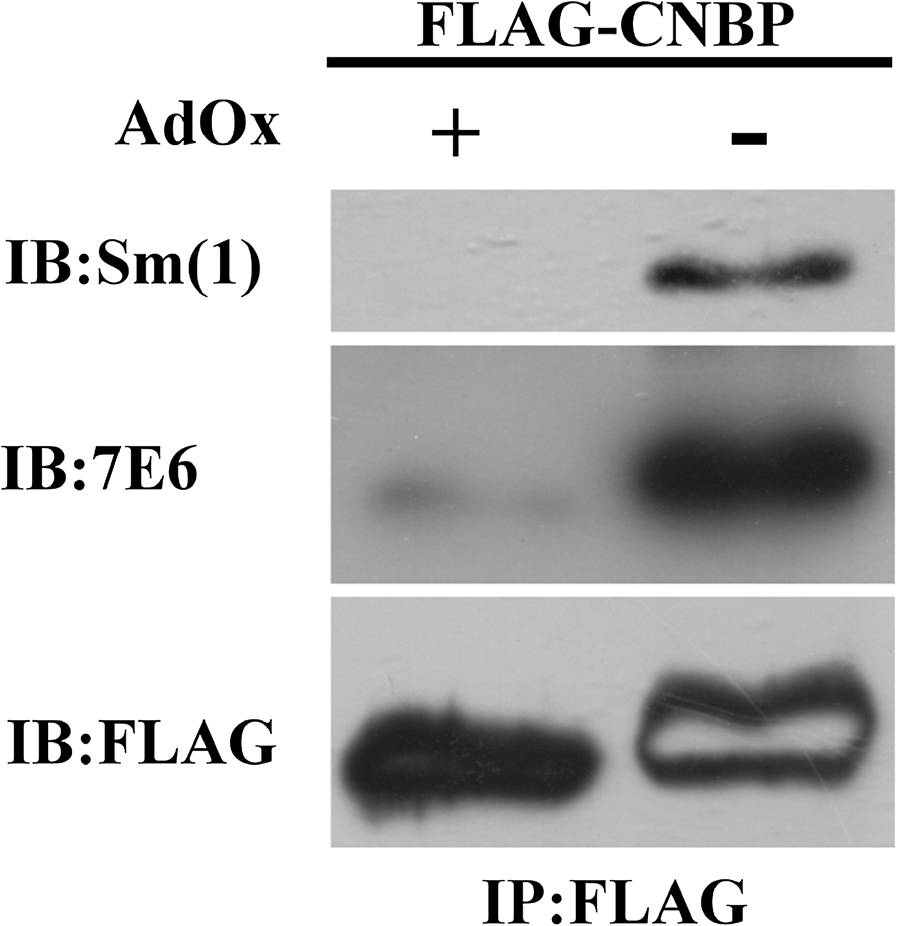
**CNBP is methylated in vivo and is recognized differentially by Sm1 due to its methylation status.** HeLa cells were transfected with pFLAG-CNBP plasmids. The FLAG-tagged proteins were immunoprecipitated by anti-FLAG agarose. Proteins eluted by the FLAG peptide were then analyzed by Western blot analyses with anti-Sm1. The blot was stripped of the interacted antibodies and then probed with a mono- and di-methylarginine-specific antibody 7E6. The same blot was stripped and then re-probed with the anti-FLAG antibody. The internal blank region of the FLAG-CNBP signal of the AdOx- sample detected by anti-FLAG was due to previous stripping.

We then prepared immunopurified FLAG-CNBP protein from transfected HeLa cells cultured in the presence or absence of AdOx. Sm1 detected FLAG-CNBP from untreated HeLa cells to a much higher extent than that from AdOx-treated cells (Figure 5). The same membrane was re-probed with a methylarginine-specific antibody 7E6. 7E6 clearly detected the FLAG-CNBP protein prepared from HeLa cells without AdOx treatment but not the CNBP protein from AdOx-treated cells (Figure 5). The results indicated that the CNBP protein is arginine methylated and the methylation level of the protein can be reduced by AdOx treatment. Re-probing of the membrane with anti-FLAG antibody showed equal loading, indicating the reduced FLAG-CNBP signal of the AdOx treated samples detected by Sm1 was not due to reduced protein level in the sample. Overall the experiments indicated that the recognition of CNBP by the specific SLE autosera Sm1 relies on arginine methylation.

### Differential recognition of CNBP by other anti-Sm positive patient sera

**Figure 6:**
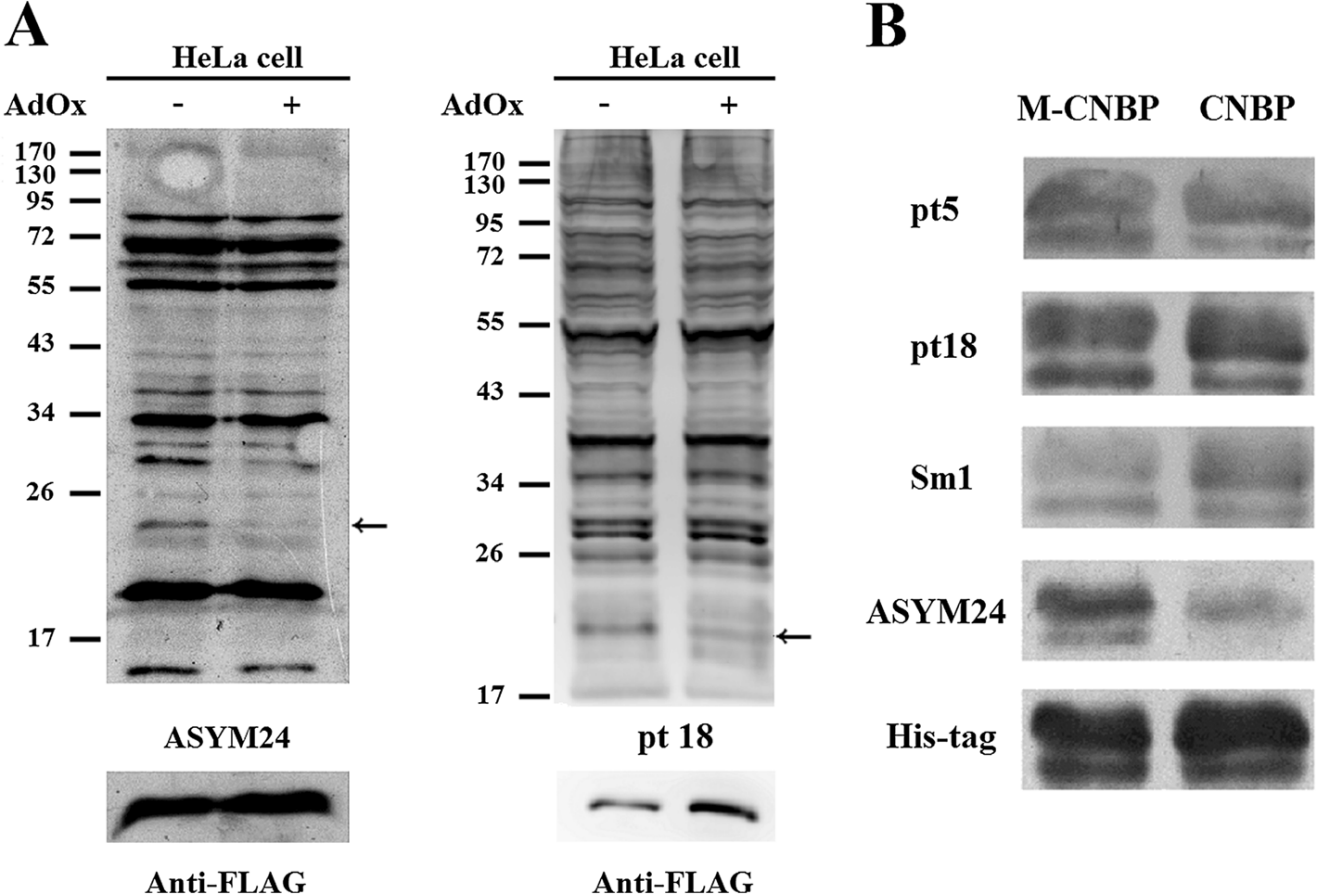
**Differential detection of CNBP protein by anti-Sm positive SLE sera.** (**A**) HeLa cell extracts prepared from cells that were transfected with the pFLAG-CNBP plasmid and treated with AdOx or not were analyzed by Western blot analyses with an aDMA-specific antibody ASYM24 (left panel) or autosera from SLE patient 18 (right panel) and re-probed with anti-FLAG antibodies. The positions of FLAG-CNBP are indicated by arrows. (**B**) (His)_6_-CNBP proteins prepared from *E. coli* cells co-expressing PRMT1(M-CNBP) or not (CNBP) were analyzed by Western blot analyses. Differential detection of (His)_6_-CNBP by autosera from patient 5, 18 and Sm1 are shown. Arginine methylation of CNBP was confirmed by the detection with ASYM24. The blot was stripped and re-probed with anti-(His)_6_ (His-tag) antibodies.

To examine whether the autoantibodies that can differentially recognize arginine methylated CNBP are present in other SLE patients, we then screened anti-Sm positive SLE patient sera for their recognition of arginine methylated CNBP. Since protein arginine methylation is rather stable, inhibition of the modification by AdOx treatment appears to be most effective for newly synthesized polypeptides [18]. Compared to other endogenous HeLa cell proteins that might have been synthesized before transfection and AdOx treatment, the transiently expressed FLAG-CNBP protein showed significant decrease of arginine methylation level upon AdOx treatment (Figure 6A). We thus used cell extracts from FLAG-CNBP transfected and AdOx treated HeLa cell extracts to screen the autosera. As for Sm1-3, we did not detect differential recognition of SmB/B′, D1 and D3 proteins by all of these patient sera. We cannot exclude the possibility that the recognition of Sm proteins was too strong to mask differential recognition by the anti-Sm antibodies. However, among the twenty Sm-positive SLE patient sera, two (patient 5 and 18) showed methylation differential recognition of CNBP. The results were outlined in Table 2. On the contrary, the pooled normal control sera did not detect the signal (data not shown).

Furthermore, we prepared arginine-methylated recombinant (His)_6_-CNBP fusion proteins from *E. coli* cells by co-transformation with plasmids expressing GST-PRMT1. We then screened Sm-positive sera for their recognition of the recombinant CNBP. We also observed deferential recognition of PRMT1-methylated CNBP by some of the autosera including Sm1 (Figure 6B). The results were summarized in Table 2. There were some discrepancies between the differential recognition of the methylated CNBP that were FLAG-tagged and expressed in HeLa cells or the (His)_6_-tagged CNBP expressed in *E. coli*.

## Discussions

Many methylarginine-containing proteins such as fibrillarin [10], hnRNPA1 [25] and Sm proteins [8] are autoantigens. However, the only direct link between protein arginine methylation and autoantibody recognition is the report by Brahms *et al.* that symmetric dimethylarginines in SmD1 and D3 are responsible for the majority of the anti-Sm recognition of the SLE patient sera [8]. In this study, we examined whether the anti-Sm-positive autosera from local SLE patients can differentially recognize the Sm proteins due to their methylation status. We did not detect differential recognition of the SmD1, D3 or B/B′ proteins under our experimental conditions (HeLa cells treated with the methylation inhibitor AdOx or not). Immunoblotting with an sDMA-specific antibody showed reduced symmetric dimethylation of SmB/B′ and SmD proteins upon AdOx treatment (data not shown), confirming that our treatment can effectively decrease protein arginine methylation. Nevertheless, differential recognition in other proteins was detected by the anti-Sm serum from typical SLE patients.

In our pilot study through proteomic analyses, we identified eight different proteins from seven putative differentially recognized spots with the Sm1 sera from a typical SLE patient. Three metabolic enzymes without known or predicted protein arginine methylation sites were identified. Among these, antibodies against α-enolase have been detected in many infectious and autoimmune diseases including SLE. The frequency of SLE patients with α-enolase Abs was around 20% and increased to about 60–80% for SLE with active renal disease [26]. Succinyl-CoA:3-ketoacid coenzyme A transferase 1 and aldehyde dehydrogenase X were both identified for spot 1. These two proteins have not been reported to be targets of autoantibodies. Two other proteins, putative beta-actin-like protein 3 along with LIM and SH3 domain protein 1, contain no known or predicted protein arginine methylation sites and have not been reported to be autoantigens.

Three proteins SFPQ, hnRNPDL and CNBP contain RG or RGG repeats typical for PRMT-modified arginine methylation [19], [20] were identified by proteomic analyses as putative candidate protein that can be differentially recognized by SLE. Splicing factors such as various SR proteins have been reported as autoantigens of SLE [27]. No report has suggested SFPQ as an autoantibody target. hnRNP DL shares high sequence similarity with hnRNPD (AUF1) and hnRNPA1. Anti-hnRNP A1 has been reported in autoimmune diseases including RA, SLE and MCTD as reviewed [22]. Anti-hnRNP D antibodies were detected in SLE and RA patients [23]. However, autoantibody to hnRNP DL has not been reported. In this study we showed that all of the anti-Sm positive sera from local SLE patients recognized recombinant hnRNPDL, indicating it to be a novel autoantigen.

The CNBP gene has been related to the human disease myotonic dystrophy type 2 (DM2) for the expansion of CCGC repeats in intron 1 [28]. CNBP has not been reported to be the target of autoantibodies. It is known to regulate the translation of ribosomal protein mRNA through binding to the terminal oligo pyrimidine (TOP) sequence in the 5^′^-UTR. Interestingly, two typical SLE autoantigens La and Ro are involved in the TOP-mRNA regulation. Both CNBP and La can bind to the 5^′^-TOP and their binding is mutually exclusive. They compete for the interacting protein Ro that is the common factor necessary for the binding of La or CNBP proteins [29]. As proteins in a similar functional complex/pathway appears to be processed and presented for antibody formation in autoimmune patients, whether CNBP is an autoantigen is an interesting issue.

We analyzed sera from anti-Sm positive patients and more than 60% can detect recombinant GST-CNBP protein expressed by *E. coli*. When we further prepared (His)_6_-tagged CNBP, all of the autosera we tested recognize the protein. CNBP thus appear to be a novel autoantigen recognized by the Sm-positive SLE patients.

We focused on the demonstration of the differential recognition of CNBP due to its methylation status. We prepared FLAG-tagged CNBP protein expressed in AdOx-treated HeLa cells. FLAG-CNBP from AdOx-treated cells was less recognized by Sm1 autosera compared to the CNBP protein expressed in untreated cells. The results confirmed that CNBP is methylated and the methylation facilitates the recognition of Sm1. However, the differential recognition of CNBP due to its methylation status is restricted to few anti-Sm positive SLE patient sera. Furthermore, as shown in Table 2, the differential recognition detected using the methylated (His)_6_-tagged CNBP expressed in *E. coli* were slightly different from the FLAG-tagged CNBP expressed in HeLa cells. For example, Sm1 significantly discriminate FLAG-CNBP expressed in HeLa cells treated with AdOx or not, but barely differentially recognize PRMT1-methylated or unmethylated (His)_6_- CNBP. As AdOx treatment inhibits all type of methylation but PRMT1 only catalyzes the formation of aDMA, the difference might be due to sDMA. In fact, FLAG-CNBP immunopurified from HeLa cell extracts could be detected by aDMA as well as sDMA-specific antibodies (Hu *et al.*, unpublished results). The results raised the possibility that some patient sera might distinguish sDMA but others aDMA-modified CNBP.

## Conclusions

In conclusion, through proteomic analyses, a few putative differentially recognized proteins were identified. Among them three proteins contain arginine methylation sites were identified by proteomic analyses. We showed that a portion of SLE patients who have anti-Sm autoantibodies can specifically recognize specific methylarginine containing proteins. Our study showed that hnRNP DL and CNBP are novel antigens for SLE patients and the recognition of CNBP might be differentiated dependent on the level of arginine methylation.

## Abbreviations

SLE: Systemic lupus erythematosus; PRMT: Protein arginine methyltransferase; sDMA: Symmetric di-methylarginines; aDMA: Asymmetric di-methylarginines; GAR: Glycine and arginine rich; hnRNP DL: Heterogeneous nuclear ribonucleoprotein; CNBP: Cellular nucleic acid binding protein; ANA: Antinuclear antibody; AdOx: Adenosine dialdehyde; 2-DE: Two-dimensional electrophoresis.

## Competing interests

The authors declare that they have no competing interests.

## Authors’ contributions

HHC: conducted the 2-D analyses and target protein identification. HHH: performed CNBP and hnRNP DL recognition experiments. YJL: constructed the hnRNP DL and CNBP clones. MCFJ: screened the anti-Sm sera with HeLa cell extracts expressing FLAG-CNBP. HMW: prepared methylated recombinant CNBP and screened the anti-Sm sera. TCH: anti-Sm sera preparation and manuscript revision. GJT: collected SLE patients and sera, applied IRB approval and manuscript revision. CL: overall design of the experiments and manuscript writing. All authors read and approved the final manuscript.
